# Evidence toads may modulate landing preparation without predicting impact time

**DOI:** 10.1242/bio.022707

**Published:** 2016-11-28

**Authors:** S. M. Cox, Gary Gillis

**Affiliations:** 1 Graduate Program in Organismic and Evolutionary Biology, University of Massachusetts, Amherst, MA 01003, USA; 2 Department of Biology, Mount Holyoke College, South Hadley, MA 01075, USA

**Keywords:** *Bufo marinus*, Active, Control, Forelimb, Landing, Torques

## Abstract

Within anurans (frogs and toads), cane toads (*Bufo marinus*) perform particularly controlled landings in which the forelimbs are exclusively used to decelerate and stabilize the body after impact. Here we explore how toads achieve dynamic stability across a wide range of landing conditions. Specifically, we suggest that torques during landing could be reduced by aligning forelimbs with the body's instantaneous velocity vector at impact (impact angle). To test whether toad forelimb orientation varies with landing conditions, we used high-speed video to collect forelimb and body kinematic data from six animals hopping off platforms of different heights (0, 5 and 9 cm). We found that toads do align forelimbs with the impact angle. Further, toads align forelimbs with the instantaneous velocity vector well before landing and then track its changes until touchdown. This suggests that toads may be prepared to land well before they hit the ground rather than preparing for impact at a specific moment, and that they may use a motor control strategy that allows them to perform controlled landings without the need to predict impact time.

## Abstract (precis)

**Summary:** Coordination of landing in humans involves using sensory feedback to anticipate impact time. In contrast, when and how toads position forelimbs suggests that they may coordinate landing without predicting impact.

## INTRODUCTION

**Fig. 1.**
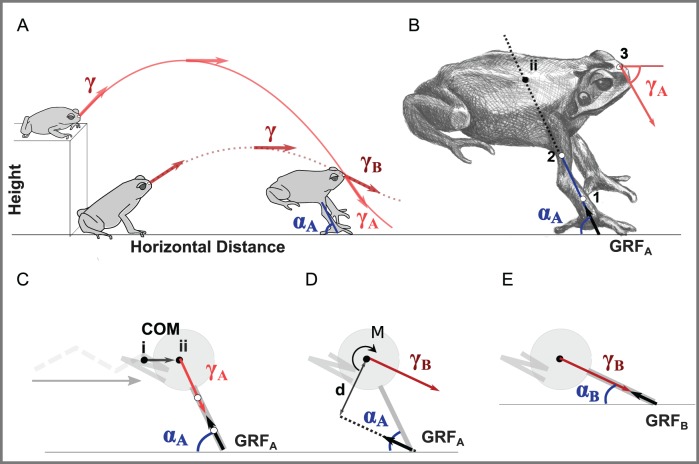
**Importance of hind- and forelimb positioning on minimizing toppling torques at landing.** (A) The velocity vector, γ, is the instantaneous tangent to the line describing the position of the snout of the toad throughout the hop. The impact angle, here shown for both a high (γ_A_) and flat (γ_B_) hop, is the instantaneous velocity vector at touchdown. Arm angle, α, was defined as the angle between the plane of the forelimb and the horizontal. Here the arms are shown with an arm angle of α_A_. (B) Close up view of the toads with points used to digitize kinematics (1-3) and the impact and arm angles for a high hop depicted. (C-E) Simplified model of toad for clarity. (C) Movement of the hind limbs from extended (dashed line) to retracted (solid line) configuration moves the center of mass (COM) anteriorly, reducing torques at impact for an impact angle, γ_A,_ and arm angle, α_A_. (D) For a more acute impact angle, γ_B_, like that depicted in the level hop in A, the same forelimb landing angle of α_A_ will result in net torques around the COM and not allow the toad to stabilize landing. (E) If forelimbs instead were positioned more anteriorly at α_B_, GRF vector could align more closely with the COM.

Toads have recently been used as a model system for understanding the biomechanics and control of landing in anurans (frogs and toads) (Gillis et al., 2014). Within anurans, cane toads (*Bufo marinus*), a group well known for jumping, perform particularly controlled landings in which they can absorb impact energy exclusively with their forelimbs (Fig. 1) before lowering their hind limbs relatively slowly to the ground (Essner et al*.*, 2010; Gillis et al*.*, 2010; Akella and Gillis, 2011; Reilly et al., 2016). In contrast, less controlled landers either collapse or topple during landing, absorbing some impact energy with other portions of their body (Essner et al*.*, 2010). The dynamic stability (*sensu* Hof et al*.*, 2005) cane toads achieve during landing requires both that the underlying musculature is prepared and sufficient to absorb the hop's energy, and that the impact forces are orientated appropriately with respect to the forelimbs. Toads prepare musculature to absorb impact by stiffening the joints through co-activation of antagonistic muscles at the elbow and wrist well before touchdown (Gillis et al*.*, 2010; Akella and Gillis, 2011; Ekstrom and Gillis, 2015). They also change forelimb configuration at impact with hop distance (Cox and Gillis, 2015), which, in part, helps to keep elbow extensors operating at lengths that minimize muscular damage (Azizi and Abbott, 2013). However, less is known about how toads control the orientation of impact forces to minimize torques at landing.

One hypothesis is that toads use hind limb retraction to control landing torques. One of the major distinctions between anurans that crash land and those that control landings is that skilled landers retract their hind limbs mid-flight (Reilly et al*.*, 2016). Azizi et al. (2014) showed that retraction of a toad's hind limbs in mid-flight moves the center of mass (COM) anteriorly and more in line with the ground reaction force (GRF) vector (Fig. 1C), minimizing torques during impact. Yet, experimental manipulations of the COM did not result in counteracting changes in hind limb retraction (Azizi et al*.*, 2014). Further, additional work has shown that variations in hind limb retraction rates in preparation for landing are not likely the result of variations in hind limb flexor muscle activity, but instead may be tied to elastic energy stored in the stretching of muscle-tendon complexes during takeoff (Schnyer et al*.*, 2014). This suggests that hind limb retraction, while important for repositioning the center of mass before landing, is a function of takeoff effort rather than landing conditions and thus may not be sufficient to control landings in varied landing conditions.

We suggest forelimb position should help orient impact forces and serve as a critical contributor to stable landings. To illustrate this, consider a toad hopping off an elevated platform and approaching landing with an impact angle *γ_A_*, and forelimbs positioned at angle *α_A_*, from the horizontal (Fig. 1A,B). For simplicity, if we model the toad morphology as a mass (body) with forelimbs as simple linear segments making ground contact at one point (Fig. 1C), at impact the GRF will be parallel to the impact angle. Azizi et al. (2014) demonstrated that the retraction of the hind limbs moves the COM anteriorly (Fig. 1C, ii to i), minimizing the moment arm of the GRF (Azizi et al*.*, 2014). However, retracting the hind limbs in this manner would not minimize torques for all impact angles. For instance, if a toad prepares to land by similarly positioning its forelimbs but approaches the ground at a more acute angle (*γ_B_*, Fig. 1D), this same body and limb configuration would now result in a net torque (Fxd) (Fig. 1D) that would topple the toad forward. In fact, at this impact angle and forelimb position, the COM would need to be well behind the toad for the animal to minimize landing torques. However, as suggested by Nauwelaerts and Aerts (2006), forelimb angle also contributes to a controlled landing. If the toad simply repositions its forelimbs to hit the ground at a more acute angle (*α_B_*, Fig. 1E), the GRF vector would again align more closely to the COM, contributing to a more controlled landing. Notice that in both cases the forelimb angle at impact mirrors the impact angle to minimize torques.

Thus, we hypothesize that toads align the forelimb landing angle (*α_TD_*) with the impact angle (*γ_TD_*). In order to test this we used high-speed video to measure body and forelimb kinematics as toads hopped off platforms of three heights, a method employed to expand the range of impact angles.

If toads do align the forelimbs to the impact angle at touchdown, our experimental method allows us to ask a second question: when does this alignment occur? How and when this is accomplished could provide insights into the control strategies at work. Specifically, we ask whether the onset or the duration of alignment significantly varies between hops off different height platforms. Aligning forelimbs a fixed duration before landing, despite hopping off different height platforms, would suggest that toads are using visual information to predict landing. Since earlier work indicated that other aspects of cane toad landing preparation may not require predictions of impact time, we hypothesize that alignment durations, but not onset times, vary significantly between hops off different platforms.

## RESULTS

### Forelimb angle versus velocity vector angle at touchdown

**Fig. 2.**
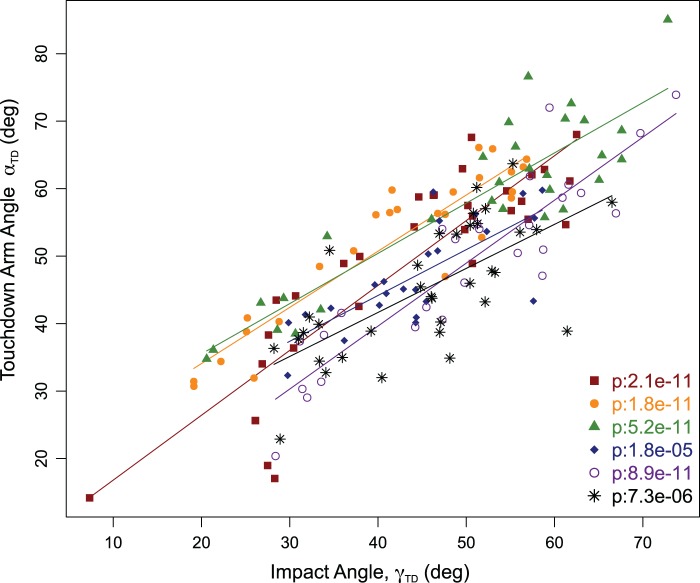
**Forelimb angle at touchdown, versus impact angle at touchdown.** Different symbol and colors reflect different animals and every symbol is a single hop. Regression lines are shown when fits are significant.

Forelimb angle at touchdown (*α_TD_*) varied significantly and linearly with impact angle (*γ_TD_*) for every animal (Fig. 2). On average the forelimb angle at touchdown (*α_TD_*) was 4.1±7.6° greater than the impact angle (*γ_TD_*). Additionally, forelimb angle at touchdown (*α_TD_*) increased with hop height [χ^2^(1):142, *P*:<1e−6]. The difference between arm angle and the impact angle (*δ_TD_*) was greatest in flat hops (Table 1). All results are reported as means of individual means±standard deviation.

**Table 1.**
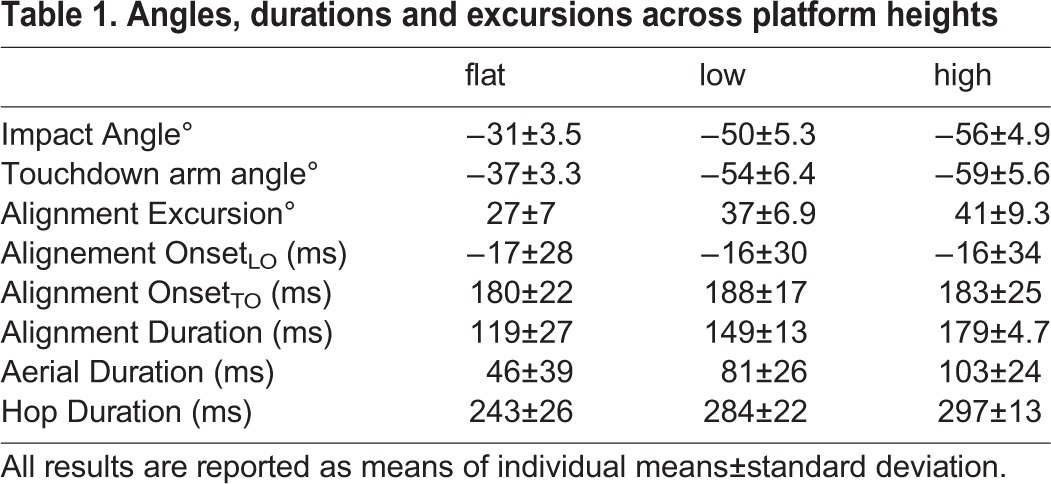
**Angles, durations and excursions across platform heights**

### Alignment patterns and timing

**Fig. 3.**
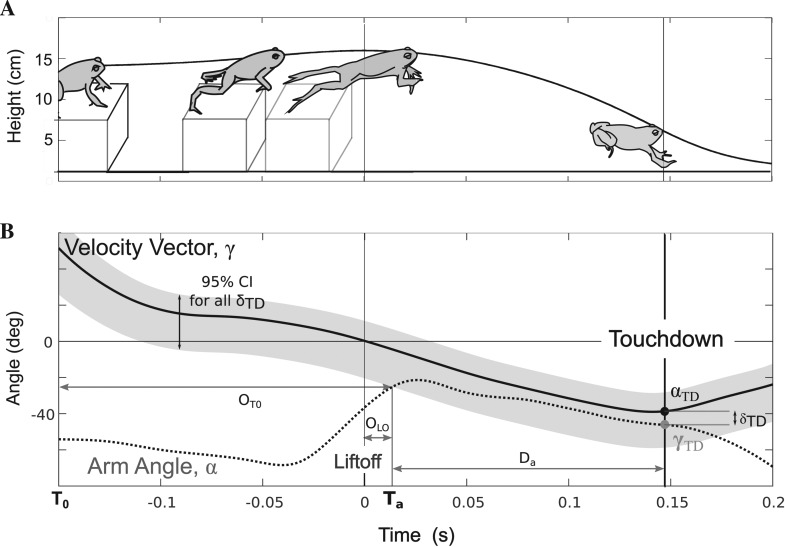
**Toad body and limb configuration, velocity vector and arm angle throughout a typical high platform hop.** (A) Toad body and limb configuration and hop height versus time. Time is zeroed at lift-off. (B) The corresponding velocity vector (*γ*, solid line) and am angle (*α*, dotted line) to the height profile depicted in A is defined as the difference between the velocity vector at touchdown (*α_TD_*) and the arm angle at touchdown (*γ_TD_*). The 95% confidence interval for *δ_TD_* is depicted in the gray region shadowing the velocity vector. For hops off all height platforms, forelimb arm angle (*δ_TD_*) typically becomes more negative during hop initiation, sharply increases near lift-off, approaching the value of the velocity vector near the crest of the hop and then tracks the velocity vector until touchdown. The time of alignment (*T_a_*) was defined as the time that the arm angle first falls within the 95% *δ_TD_* confidence interval. Alignment onset (*O_a_*) is the duration from liftoff to (*T_a_*) while alignment duration (*D_a_*) is the time difference between *T_a_* and touchdown.

The difference between the velocity vector and arm angle (***δ***) followed a typical pattern throughout hops for all platform heights (Fig. 3). At hop onset, ***T***_0_, arms were positioned 75±6.9° less the velocity vector. This difference increased during hop initiation as toads raised the trunk to takeoff, reaching an average maximum ***δ*** of 89±8.9°. Afterward, ***δ*** steadily decreased, aligning with the velocity vector (±15° of ***α***) 184±3.8 ms after hop onset and 16±0.8 ms before lift-off. Toads maintained alignment between the arm angle and velocity vector over an excursion of 35±7.2° (Table 1).

**Fig. 4.**
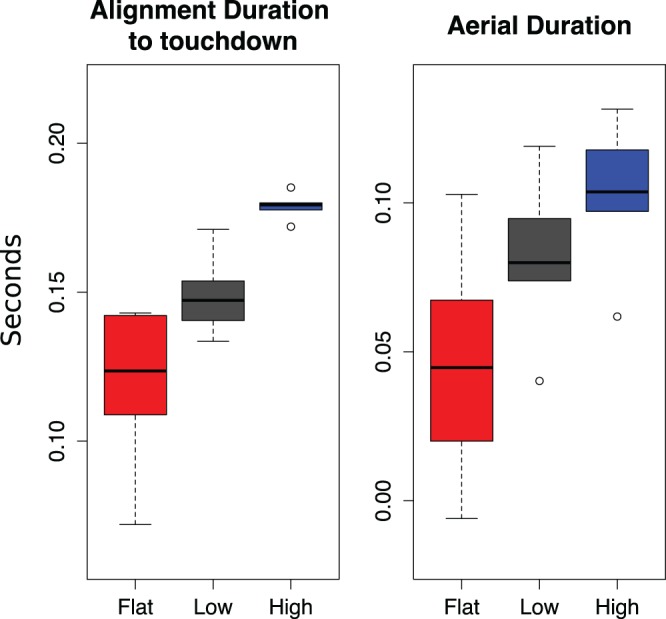
**Alignment and aerial duration by treatment.** Boxplots are from means of individual means in each treatment. As aerial duration increases, alignment duration increases.

**Fig. 5.**
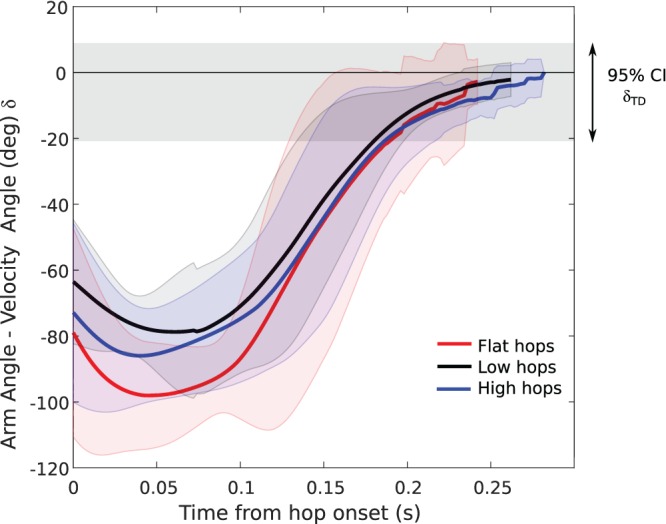
**The difference between arm angle and velocity vector (*δ*) from hop initiation to average touchdown across all hops by platform treatment.** Solid lines depict the mean and the shaded regions show the 95% CI around that mean for each treatment. Flat hops are shown in red, low hops in black and high hops in blue. The 95% CI for *δ_TD_* is depicted by the shaded gray region around zero. Across all platforms, arm position does not significantly vary during the hop. Toads align forelimbs at approximately the same time from hop initiation, maintaining alignment longer in longer hops.

The onset of alignment of forelimbs from toad lift-off or hop initiation did not significantly vary between hops off different platforms (O_LO_: χ^2^:0.16, *P*: 0.92, O_T0_: χ^2^:0.88, *P*: 0.65). During the aerial phase, toads maintained alignment between the arm angle and velocity vector until touchdown (98.7% of hops continually maintaining alignment) resulting in alignment durations that significantly varied between platforms (χ^2^:28, *P*: 6.7e−07, Table 1) and increased with platform height (Table 1, Fig. 4, χ^2^:28, *P*: 6.7e−07). Thus, toads did not vary the timing of forelimb alignment across different platform heights. Instead, toads aligned forelimbs with the velocity vector at or slightly before lift-off and tracked the velocity vector until touchdown across all treatments (Fig. 5).

## DISCUSSION

As we hypothesized, toads align forelimbs with the impact angle at touchdown. Our results are consistent with Nauwelaert's model of landing in *Rana esculenta* (Nauwelaerts and Aerts, 2006), in which peak ground reaction forces were minimized by positioning forelimbs more vertically during hops of greater height (in our terms, with a greater forelimb angle). These results also align with the requirements for dynamic stability during landing put forth by Patton et al. (1999), which suggested hops with higher horizontal velocities could be stabilized at impact by positioning the landing limbs further forward of the COM (in our terms, with a smaller forelimb angle). Notice that both models' predictions (Pai and Patton, 1997; Nauwelaerts and Aerts, 2006) are just special cases of the more general framework we propose here where the forelimb angle is aligned with impact angle, as defined by both its horizontal and vertical components. Our framework is more in line with collision-based analyses of locomotion (Ruina et al*.*, 2005; Lee et al*.*, 2011), where the collision angle is defined as the angle between the impact angle and the line perpendicular to the GRF. While in running the cost of transport is decreased by minimizing collision angles (Ruina et al*.*, 2005; Lee et al*.*, 2011, 2013; Gutmann et al*.*, 2013), controlled landing demands the opposite, namely increasing the amount of energy lost by maximizing the collision angle via aligning the GRF and the impact angle.

In our framing of the analysis, rather than measuring the complex kinetics associated with landing, we simplified the toad morphology into a mass (body) with linear segments (forelimbs) (Fig. 1) making ground contact at one point, and assumed the GRF vector aligns with the velocity vector of the COM at impact. This means we also did not account for any angular acceleration or active muscular contributions during impact that could influence landing kinetics and kinematics; however, we justify these simplifications since our primary focus was on how toads align forelimbs before impact rather than exploring the more complex kinetics after impact. A more thorough analysis of GRF and muscular activation patterns during landing would allow us to check the validity of these assumptions, and explore the relationship between minimizing joint moments and torque around the body's center of mass.

We found that toads align forelimbs with the velocity vector well before impact and track the changing velocity vector throughout the aerial phase. By matching forelimb and velocity angles early in the hop and tracking the velocity vector until impact, the toad is essentially prepared to land at any time over a long interval rather than preparing for impact at a specific moment. This mirrors the control strategy toads use to produce the distance-dependent increases in elbow extension to provide greater breaking distances after impact in longer hops (Cox and Gillis, 2015). Toads begin to extend their elbows at roughly the same time and at the same rate in all hops, and longer hops simply provide more time to reach more extended configurations (Cox and Gillis, 2015). In both cases, toad forelimb kinematics in preparation for impact seem to modulate forelimb position and configuration so that they are prepared to land well before the time of impact, eliminating the need to predict when that impact will happen.

If toads are not using predictions of impact time to control landing preparation, it leaves open the question of whether they require a trigger to start landing preparation and, if so, what the trigger might be. While this and an earlier study of forelimb kinematics are consistent with a control strategy for landing preparation that does not begin landing preparation based on predictions of impact time (Cox and Gillis, 2015), cane toads have also been shown to begin to brace underlying musculature in preparation for landing later in longer hops (Gillis et al*.*, 2010, 2014; Akella and Gillis, 2011), implying some prediction of hop duration and tuning of the landing timing preparation to hop conditions. Yet, it would be surprising if toads only used predictions of impact time to modulate forelimb muscle activation and not kinematics in preparation for landing. One possible explanation could be that toads may not be predicting the specific time of impact from integration of the optic flow, as is common in many mammals and birds (McKinley and Smith, 1983; Liebermann and Goodman, 1991; Santello et al*.*, 2001; Santello, 2005; Gordon et al*.*, 2015), but are instead using inherently distant dependent vestibular cues to initiate portions of the landing sequence. For instance, if toads begin to brace for impact at the time of maximum hop height, muscles would tend to be activated later in longer hops, exhibiting the distance dependence observed without the need for specific temporal predictions. Distinguishing whether toads are predicting impact time or using vestibular cues to initiate impact preparation is difficult, since the two are highly correlated in hops on flat surfaces. Studies exploring pre-landing electromyography (EMG) activity of toads hopping off platforms, much like our experimental setup, may be able to pull apart vestibular cues from hop duration enough to shed some light on the triggers toads use to initiate impact preparation in the underlying musculature.

Another question left unanswered by this study is whether toads are actively positioning forelimb angle to match the velocity vector. While the close alignment between the forelimb angle and the velocity vector suggests active control, it could also be a passive consequence of hop dynamics or elastic energy storage. Either control strategy would be consistent with earlier work on cane toad landing control which appears to consist of some active (Cox and Gillis, 2016) and some passive components (Azizi et al*.*, 2014; Schnyer et al*.*, 2014). If arm angle were actively controlled, it would be consistent with the accumulating evidence that toads may prioritize vestibular or proprioceptive feedback over visual feedback to prepare for landing (Gillis et al*.*, 2014; Cox and Gillis, 2016). Again, additional work that incorporated EMG data could shed more light on these questions.

In summary, cane toads are known to perform controlled landings hopping on flat surfaces. Here we see that they can also accommodate varying terrain, achieving landings from heights 2-3 times their body height. Previous work shows that this appears to be accomplished by coordinating three components of impact preparation: (1) positioning the forelimbs to hit the ground first by protracting and abducting the humeri (Cox and Gillis, 2015), (2) preparing and bracing for impact by extending the elbows and activating underlying musculature to stiffen the joint (Gillis et al*.*, 2010; Akella and Gillis, 2011), and (3) controlling torques during the landing in part by retracting the hind limbs (Azizi et al*.*, 2014). Our work here adds another important element of controlled landing, namely that toads also align the forelimbs with the impact angle. Further, we found that they align forelimbs well before landing and then track the velocity vector through the aerial phase of the hop. This provides additional evidence that toads may modulate landing preparation without relying on visual information to predict impact time.

## MATERIALS AND METHODS

### Animals

Six female adult *B. marinus* (63-170 g) were obtained from a commercial supplier and housed in large plastic containers in groups of two to four in a room maintained at ∼24°C with a 12 h light:12 h dark cycle. Water was always available and they were fed a diet of crickets several times a week. All experimental work was approved by Mount Holyoke College Institutional Animal Care and Use Committee (IACUC), MA, USA.

### Jumping trials

The toad's limbs were marked at the wrist (Fig. 1B, point 1) and midway along the humerus (Fig. 1B, point 2) to characterize forelimb angle in relation to the horizontal, and a point near the tip of the animal's snout was used to quantify the instantaneous velocity vector of the toad's body (Fig. 1, point 3). The mid-humeral point was used because more proximal points were often obscured in at least one camera view by the large parotid glands on the animal’s dorsal surface. Animals were hopped in a rectangular glass tank (89×43×43 cm) off platforms of three heights in a random order (flat: 0 cm, low: 5 cm, high: 9 cm) lined with rough felt to provide purchase during takeoff (6-12 hops for each of 6 individual toads in each condition; 26-35 hops for each toad, 173 hops total). Hops were recorded with two high-speed cameras (Fastec HiSpec, San Diego, CA, USA) at 500 fps (1280×1024 pixels). Videos were calibrated with a 64 point calibration cube and marker points were digitized with Matlab software (Hedrick, 2008) as in (Cox and Gillis, 2015). Animals sometimes hopped downwards off the high platform and to maintain consistency across treatments, hops were only included in analysis if at hop initiation (T_0_), when the toad's velocity first reached 5 cm/s, there was an upward vertical velocity component.

### Data analysis

All video sequences were analyzed to identify the onset of movement, and time of lift-off and touchdown for each hop. 3D coordinates were smoothed with a quintic spline interpolation. Forelimb angle (*α*) was calculated as the angle between the line through points 1 and 2 (Fig. 1B), and the horizontal such that forelimbs held parallel to the ground would have an angle of 0°. Velocity vector angle (*γ*) was the instantaneous tangent to the line describing the position of the tip of the animal's snout through time (Fig. 1C). The difference between the velocity vector and forelimb angle (*δ*) was defined as *γ – α*. The difference between the impact angle and forelimb angle at touchdown (*δ_TD_*) was taken to be *δ* at first manus touch. Hop initiation, T_0_, was defined as the first time the velocity of the toad was greater than 5 cm/s.

To determine whether the forelimb angle aligned with the impact angle at touchdown, the forelimb angle at touchdown (*α_TD_*) was regressed against the impact angle (*γ_TD_*) for each toad (Fig. 2). Additionally, we used a linear mixed model (Bates et al*.*, 2015) to analyze how forelimb angle at touchdown varied with impact angle across all toads. We compared full models with individual toad as a fixed factor and impact angle as a fixed effect to the null model with no fixed effect. The *P*-value for the full model was computed with a likelihood ratio test between the full and reduced model.

To test our second hypothesis that forelimb alignment duration, but not onset time, changes with platform height, we first defined the alignment of forelimbs with velocity vector. The absolute value of the difference between forelimb angle and the velocity vector (*δ*) followed a stereotypical pattern across all hops, first increasing, reaching a maximum, and then decreasing (Fig. 3). We defined the time of forelimb alignment, T_a_ (Fig. 3), as the time that *δ* first entered the 95% confidence interval for *δ_TD_* (gray region Fig. 4). We compared the time from hop initiation (*OT*_0_) and lift-off to alignment (*O_LO_*) and the duration from alignment to touchdown (*D_a_*) between the three platform treatments (Fig. 3) by fitting each with two mixed linear models: a null model with no fixed effect, and a full model with platform treatment (flat, low and high) as a fixed effect. We similarly analyzed the relationship between alignment duration and hop duration. In all models, individual toads were included as random effects. Again, the *P*-value for each model was computed with a likelihood ratio test between the full and reduced model. All analyses were conducted in R (R Core Team, 2015).
